# 

**Published:** 1883-01-20

**Authors:** 


### TABLE OF CONTENTS.

ORIGINAL AND SELECTED ARTICLES.

Acute Rheumatism, by G. G. Roy, M. D., Professor of Materia Medica in The Southern Medical College, Atlanta, Ga. 1. Treatment of Iritis, by A. G. Hobbs, M.D., Professor of Ophthalmology. Extract from a clinical lecture at the Southern Medical College, Atlanta, Ga., 4. Ingrowing Toe-Nail, by R C. Word, M.D., Professor of Physiology in the Southern Medical College, Atlanta, Ga., 6. Irrigation of the Colon, by Chas. W. Dulles, M. D., Surg. Rep. to the Hospital of the University of Pennsylvania, 7. Three Cases of Nasal Alimentation, by D. N. Rankin, A. M., M.D., of Alleghany City, Pennsylvania, 9. Treatment for Tape-Worm, from Squibb's Ephemeris, 11. Agoraphobia—A Contribution to Clinical Medicine, by L. T. Potter, B. L., M.D., Clinical Lecturer on Diseases of the Chest, South Side Dispensary, Chicago, Illinois, 14. Medico-Chirurgical College, Service of F. Le Sieure Weir, M. D., Clinical Professor of Dermatology. Reported by Dr. H. B. Nightingale, Chief of Clinic, 15.

ABSTRACTS AND GLEANINGS.

Idiocy, 20. Aspiration of Bladder, 21. Ergot in Cancer, 22. Subcutaneous Injection in Pueperal Convulsions, 25. Clinical Remarks on Boracic Acid. 26. The Latest about Bacteria, 27. Angina Pectoris—Death; Naphthaline as an Antiseptic. 28. Bacteria of Syphilis; New Anæsthetics; Abortive Treatment of Facial Erysipelas; According to, 29. Albuminuria in Phthisis; Small Doses; The Development of Vaccinia from Variola, 30.

SCIENTIFIC ITEMS.

Misers; Vanadium; Submarine Work; Australian Big Trees, 31. Hurricanes; The Moon and the Weather, 32.

PRACTICAL NOTES AND FORMULÆ.

Treatment of Malarial Fever; Dr. Gross' Neuralgic Pills, 33. Hypodermic Use of Quinine; Emmenagogues; Nervine and Anti-Spasmodic, 34. Boracic Acid Cotton; Iodoform in Chronic Pulmonary Affections, 35.

EDITORIALS AND MISCELLANEOUS.

Editorial Notices, 36. Death of Prof. Draper; College of Medical Practitioners, St. Louis; Grave Robbing; Taxing Physicians, 37. Medical Society of Tennessee, 38. The New Year and the Physician; Book Notices, 39. Receipted; Special Notices, 40.

